# Multi-omics approach reveals CCND1, GABPA, HIF1A, and SOX6 as key regulators and prognostic markers in heart failure

**DOI:** 10.1186/s41065-025-00536-y

**Published:** 2025-08-16

**Authors:** Ping He, Lang Deng, Kaijie Wu

**Affiliations:** 1 Fifth School of Clinical Medicine of Zhejiang, Huzhou Central Hospital, Chinese Medical University, Huzhou, 313000 Zhejiang China; 2 Huzhou Central Hospital, Affiliated Central Hospital of Huzhou University, Huzhou, Zhejiang, 313000 China; 3 Huzhou Wuxing Hospital of Traditional Chinese and Western Medicine, Huzhou, 313000 Zhejiang China

**Keywords:** Heart failure (HF), Differentially expressed genes (DEGs), Hub genes, Risk model, Immune cell infiltration

## Abstract

**Introduction:**

Heart failure (HF) is a progressive condition with complex molecular mechanisms. This study aims to identify potential biomarkers and therapeutic targets by analyzing differentially expressed genes (DEGs) in HF patients, exploring the roles of hub genes, and developing a risk model for predicting disease progression.

**Methodology:**

We cultured five human HF cell lines and five normal coronary cardiomyocyte cell lines. Gene expression datasets were retrieved from the Gene Expression Omnibus (GEO) database and analyzed using limma. Protein-Protein Interaction (PPI) networks were constructed with STRING, and immune cell infiltration was analyzed using CIBERSORT. A risk model was built using LASSO regression. Drug screening was performed via CMap, and overexpression studies of CCND1 and HIF1A were conducted in AC16 and SEKHEP1 cells via cell proliferation, colony formation, and wound healing assays.

**Results:**

We identified 182 common DEGs associated with HF. Hub genes CCND1, GABPA, HIF1A, and SOX6 were central in the PPI network. LASSO regression established a risk model linked to disease progression. Immune infiltration analysis revealed altered immune cell profiles in HF. The miRNA-mRNA network showed interactions of hsa-miR-93-5p, hsa-miR-802, hsa-miR-199a-5p, and hsa-miR-203a-3p with hub genes. Overexpression of CCND1 and HIF1A in cell lines impaired proliferation, colony formation, and migration, implicating their role in HF pathophysiology.

**Conclusion:**

CCND1, GABPA, HIF1A, and SOX6 may serve as biomarkers for HF. Our findings provide valuable insights into immune infiltration, miRNA regulation, and the identification of therapeutic targets for HF management. These results highlight the role of gene regulation in HF progression and may guide future therapeutic interventions.

**Supplementary Information:**

The online version contains supplementary material available at 10.1186/s41065-025-00536-y.

## Introduction

Heart failure (HF) is a complex, multifactorial syndrome characterized by the heart’s inability to pump blood efficiently to meet the body’s metabolic demands [1–3]. It is a leading cause of morbidity and mortality worldwide, affecting approximately 64 million people globally [4, 5]. The prevalence of HF has been steadily increasing due to the aging population, improved survival from cardiovascular diseases such as myocardial infarction, and other risk factors like hypertension and diabetes mellitus [6, 7]. The condition imposes a significant burden on healthcare systems, with high hospitalization rates, long-term care requirements, and associated healthcare costs [6, 8, 9]. Despite advances in medical therapies and device-based interventions, the prognosis for HF patients remains poor, with many individuals experiencing progressive disease, hospitalization, and ultimately, death [10, 11]. Gaining a deeper understanding of the molecular mechanisms that drive HF progression is essential for identifying potential biomarkers and therapeutic targets that could enhance clinical outcomes and alleviate the disease burden.

HF presents a major public health challenge, particularly in developed nations, where it is among the most common causes of hospitalization in individuals aged over 65 [12–14]. However, despite the widespread prevalence of HF, our understanding of its molecular underpinnings is still incomplete [13, 15]. Several pathophysiological mechanisms, including inflammation, fibrosis, myocardial injury, and metabolic dysfunction, contribute to HF, but the specific genes that regulate these processes remain poorly understood [16, 17]. In parallel, the field has witnessed rapid progress in the integration of multi-omics data—including transcriptomics, proteomics, epigenomics, and metabolomics—to construct a more holistic view of HF biology [18–20]. Integrative bioinformatics strategies such as weighted gene co-expression network analysis (WGCNA), machine learning-based feature selection, and network-based modeling have enhanced the ability to identify disease-driving genes and pathways [21, 22]. For instance, a study by Cao et al. utilized combined transcriptomic and proteomic analysis of failing myocardium to uncover novel regulators of myocardial remodeling [21]. Similarly, the Myocardial Applied Genomics Network (MAGNet) project has integrated RNA-seq, miRNA, and methylation data to prioritize hub genes involved in dilated cardiomyopathy [23]. Tools like iCluster, MOFA (Multi-Omics Factor Analysis), and DIABLO (from the mixOmics R package) have been widely applied to integrate and interpret multi-layered omics data in cardiovascular studies [24]. These approaches allow for cross-validation across omics layers, improving robustness and reducing false positives [25]. Yet, there exists a gap in our knowledge regarding the identification and validation of key hub genes that play a central role in the pathogenesis of HF. These hub genes are critical in regulating various pathways that contribute to HF progression and could serve as biomarkers for early detection or therapeutic targets.

Over the years, several studies have attempted to identify key genes associated with HF using genomic and transcriptomic approaches [26]. Initial efforts focused on identifying differentially expressed genes (DEGs) in heart tissue samples obtained from HF patients and animal models of the disease [27–29]. These studies have revealed a variety of genes involved in different biological processes, including cell cycle regulation, apoptosis, oxidative stress response, and immune modulation. One such study utilized microarray data from the GEO repository to identify hub genes in HF. They found that several genes related to inflammatory pathways and cardiac remodeling, including TNF, IL6, and MMP9, were differentially expressed in HF patients [30]. These genes were implicated in driving the pathological changes in the myocardium, such as fibrosis and cardiomyocyte apoptosis. Additionally, another employed network-based analysis to identify hub genes in HF using Gene Set Enrichment Analysis (GSEA) and protein-protein interaction (PPI) networks. Their work identified TP53, VEGFA, and CXCL12 as key regulatory genes involved in inflammation and angiogenesis in HF [31]. In another study, a comprehensive analysis of transcriptomic data from HF patients and animal models revealed several key genes, such as MYC, CCND1, and HIF1A, which were found to play central roles in regulating cardiac hypertrophy and fibrosis [32, 33].

Despite ongoing efforts, there is still no consensus on the key hub genes and their molecular mechanisms in HF. Many studies have focused on isolated gene expression changes without integrating bioinformatics tools to identify critical regulatory genes within the broader molecular network. This study aims to fill this gap by using in silico and in vitro analyses to identify and validate hub genes involved in HF. By analyzing gene expression data from the Gene Expression Omnibus (GEO) repository, we aim to uncover key regulators of HF’s molecular pathways, which could lead to the development of more targeted therapies beyond current treatments like ACE inhibitors and beta-blockers.

## Methodology

### Data acquisition, identification of DEGs and hub genes in HF patients

Firstly, we retrieved three gene expression datasets of HF patients from the GEO database (https://www.ncbi.nlm.nih.gov/geo/) [34], including GSE21610, GSE57338, and GSE5406. The GEO database is a public repository that provides access to high-throughput gene expression data. For each dataset, raw were downloaded. When raw CEL files were accessible, background correction, log2 transformation, and quantile normalization were performed using the affy or oligo packages in R. For all datasets, probe IDs were mapped to gene symbols based on the corresponding platform annotation files, and when multiple probes corresponded to the same gene, their average expression value was used. To minimize non-biological variability, we applied batch effect correction using the sva package’s ComBat function, which is widely used to adjust for batch effects in high-throughput datasets. The limma package [35, 36] in R was used to analyze the DEGs between the HF and normal control samples. limma is an established tool for analyzing differential gene expression in microarray and RNA-seq data, allowing for robust identification of significant DEGs across conditions. For DEG identification, we applied an adjusted *p*-value threshold of adj. *p* < 0.05 (Benjamini-Hochberg method) to control the false discovery rate (FDR), and a log2 fold-change cutoff of |logFC| >1 to ensure biological relevance. These thresholds were selected based on commonly accepted standards in transcriptomic studies to balance sensitivity and specificity, ensuring identification of genes with both statistically significant and biologically meaningful expression differences. To investigate the functional relationships between the identified DEGs, we constructed a Protein-Protein Interaction (PPI) network using the STRING database (https://string-db.org/) [37, 38]. STRING is a widely used bioinformatics tool that provides data on protein interactions derived from both experimental studies and computational predictions. The PPI network was then analyzed using Cytoscape software (https://cytoscape.org/) [39], which is used for visualizing molecular interaction networks. The CytoHubba application [39] within Cytoscape was employed to assess the centrality of the proteins in the network, identifying the hub genes based on their degree of connectivity within the network.

### Cell culture

We purchased 5 human HF cell lines (AC16, SEKHEP1, H9C2, iPSC-CM, and ACM) and 5 normal human coronary cardiomyocytes cell lines (HCM, HCAEC, hCMSCs, hCPCs, and iPSC-CPCs) from ATCC, Lonza, and Cellular Dynamics International, USA. For cell culture, we used Dulbecco’s Modified Eagle Medium (DMEM) (Thermo Fisher Scientific, #11965092), Fetal Bovine Serum (FBS) (Thermo Fisher Scientific, #16000044), and Penicillin-Streptomycin Solution (Thermo Fisher Scientific, #15140122) to maintain the cell lines. Cells were incubated at 37 °C in a humidified atmosphere with 5% CO₂.

### Reverse transcription quantitative polymerase chain reaction (RT-qPCR) analysis

Total RNA was extracted using the RNeasy Mini Kit (Qiagen, #74104) [40]. cDNA was synthesized from 1 µg RNA using the High-Capacity cDNA Reverse Transcription Kit (Thermo Fisher, #4368814). RT-qPCR was performed with PowerUp SYBR Green Master Mix (Thermo Fisher, #A25742) on a QuantStudio 6 Flex System to quantify CCND1, GABPA, HIF1A, SOX6, AKT, PTEN, and VEGF expression. GAPDH served as the internal control, and relative expression was calculated using the 2^^(−ΔΔCT)^ method [41]. Primer sequences used are listed below.

GAPDH-F 5’-ACCCACTCCTCCACCTTTGAC-3’,

GAPDH-R 5’-CTGTTGCTGTAGCCAAATTCG-3’

CCND1-F: 5’-TCTACACCGACAACTCCATCCG-3’

CCND1-R: 5’-TCTGGCATTTTGGAGAGGAAGTG-3’

GABPA-F: 5’-CTGCTGCACTGGAAGGCTATAG-3’

GABPA-R: 5’-GGTGAGGTCTATATCGGTCATGC-3’

HIF1A-F: 5’-TATGAGCCAGAAGAACTTTTAGGC-3’

HIF1A-R: 5’-CACCTCTTTTGGCAAGCATCCTG-3’

SOX6-F: 5’-GCCTAAGTGACCGTTTTGGCAG-3’

SOX6-R: 5’-GGCATCTTTGCTCCAGGTGACA-3’

AKT-F: 5’-TGGACTACCTGCACTCGGAGAA-3’

AKT-R: 5’-GTGCCGCAAAAGGTCTTCATGG-3’

PTEN-R: 5’-TGAGTTCCCTCAGCCGTTACCT-3’

PTEN-F: 5’-GAGGTTTCCTCTGGTCCTGGTA-3’

VEGF-R: 5’-TTGCCTTGCTGCTCTACCTCCA − 3’

VEGF-F: 5’-GATGGCAGTAGCTGCGCTGATA-3’

### Risk model construction

The least absolute shrinkage and selection operator (LASSO) [42] proportional hazards regression model was built using the GLMNET package to derive the genetic risk coefficients, and patients were classified into high-risk and low-risk groups.

### Immune cell infiltration analysis

The CIBERSORT package [43] was utilized to conduct an immune infiltration analysis of HF, evaluating the relative abundance of immune and stromal cells in HF compared to controls. This analysis aimed to gain a deeper understanding of immune infiltration changes during HF progression. Additionally, Pearson correlation analysis was performed to identify immune cells strongly associated with the hub genes.

### PPI network construction and gene enrichment analysis

To investigate the interactions and functional enrichment of hub genes, we began by constructing PPI networks. The STRING database (https://string-db.org/) [37, 44] was used to identify the interactions among the hub genes. To further understand the biological relevance of the hub genes, we performed gene enrichment analysis of their binding partners using the DAVID tool (https://david.ncifcrf.gov/) [45, 46]. DAVID provides functional annotation tools that help in understanding the biological roles of gene lists. Through this analysis, we gained insight into the cellular components, molecular functions, biological processes, and KEGG pathways associated with the hub genes and their interacting partners.

### Expression validation analysis of hub genes

To validate the expression of the hub genes, mRNA expression levels of CCND1, GABPA, HIF1A, and SOX6 were first analyzed using data from the GSE57345 dataset. This dataset, available from the GEO database (https://www.ncbi.nlm.nih.gov/geo/) [34], contains gene expression profiles of HF and normal control samples.

Western blotting was performed to assess CCND1, GABPA, HIF1A, and SOX6 protein levels in 5 HF and 5 control cell lines. Equal protein amounts were separated by SDS-PAGE, transferred to PVDF membranes (Thermo Fisher, #88518), and probed with primary antibodies (anti-CCND1, #CF804673; anti-GABPA, #MA5-15419; anti-HIF1A, #MA1-516; anti-SOX6, #PA5-81994; anti-GAPDH, #MA1-16757). HRP-conjugated secondary antibodies (#31460) and ECL substrate (#34095) were used for detection.

### miRNA-mRNA network construction and analysis

To investigate the potential regulatory role of miRNAs in regulating the expression of hub genes in HF, we used the Human TargetScan database (https://www.targetscan.org/) [47]. This database provides predictions of miRNAs that may target the 3’ untranslated regions (UTRs) of genes. Among the multiple miRNAs predicted for each gene, we selected the top miRNA based on the highest context score, which indicates the strength of miRNA binding. Other predicted miRNAs were excluded due to low binding affinity (indicated by lower context scores). The selected miRNAs—hsa-miR-93-5p for CCND1, hsa-miR-802 for GABPA, hsa-miR-199a-5p for HIF1A, and hsa-miR-203a-3p for SOX6—were thus chosen for their strong predicted binding affinity and biological relevance.

RT-qPCR was performed to assess hsa-miR-93-5p, hsa-miR-802, hsa-miR-199a-5p, and hsa-miR-203a-3p expression in 5 HF and 5 control cell lines. Total RNA was extracted using the RNeasy Mini Kit (Qiagen, #74104). miRNA quantification was carried out using TaqMan MicroRNA Assays (Thermo Fisher, #4427975) and TaqMan Universal PCR Master Mix (#4440040) on a QuantStudio 6 Flex System. Relative expression was calculated using the 2^^(−ΔΔCT)^ method, normalized to U6 snRNA.

### Screening of potential therapeutic drugs

The CMap database contains 1,309 compounds and expression data for over 7,000 human genes [48]. The hub genes were uploaded to CMap, and negatively correlated small molecules were identified using the criteria of *p* < 0.0001 and a mean value < 0.4.

### Overexpression of CCND1 and HIF1A in AC16 and SEKHEP1 cells

To induce overexpression of CCND1 and HIF1A in AC16 and SEKHEP1 cell lines, we used expression vectors. The pcDNA3.1 vectors containing the coding sequences for CCND1 and HIF1A were transfected into the cells using Lipofectamine 3000 Transfection Reagent (Thermo Fisher Scientific, #L3000015) according to the manufacturer’s protocol.

### Cell proliferation assay

Cell proliferation was measured using the Cell Counting Kit-8 (CCK-8) assay (Thermo Fisher Scientific, #K1018).

### Colony formation assay

For the colony formation assay, AC16 and SEKHEP1 cells were seeded in 6-well plates at a density of 500 cells per well. After 2–3 weeks of incubation at 37 °C with 5% CO₂, colonies were fixed with 4% paraformaldehyde for 15 min and stained with crystal violet solution for 30 min. Colonies were counted manually, and the number of colonies formed was recorded.

### Wound healing assay

To assess cell migration, the wound healing assay was performed. AC16 and SEKHEP1 cells were seeded in 6-well plates and grown to confluence. A sterile pipette tip was used to create a wound in the cell monolayer, and the cells were then washed with PBS to remove any detached cells. The cells were cultured in serum-free DMEM and observed at 0 and 24 h using a phase contrast microscope. The wound closure was measured and calculated using ImageJ software (NIH).

### Statistical analysis

Data were expressed as mean ± standard deviation (SD). Statistical significance was assessed using Student’s t-test for comparisons between two groups. For multiple comparisons, one-way analysis of variance (ANOVA) was applied, followed by Tukey’s post-hoc test. P*-value < 0.05, P**-value < 0.01, and P***-value < 0.001 were considered statistically significant. All statistical analyses were performed using GraphPad Prism 8 (GraphPad Software, Inc., USA).

## Results

### Data acquisition, identification of DEGs and hub genes in HF patients

**Fig. 1:**
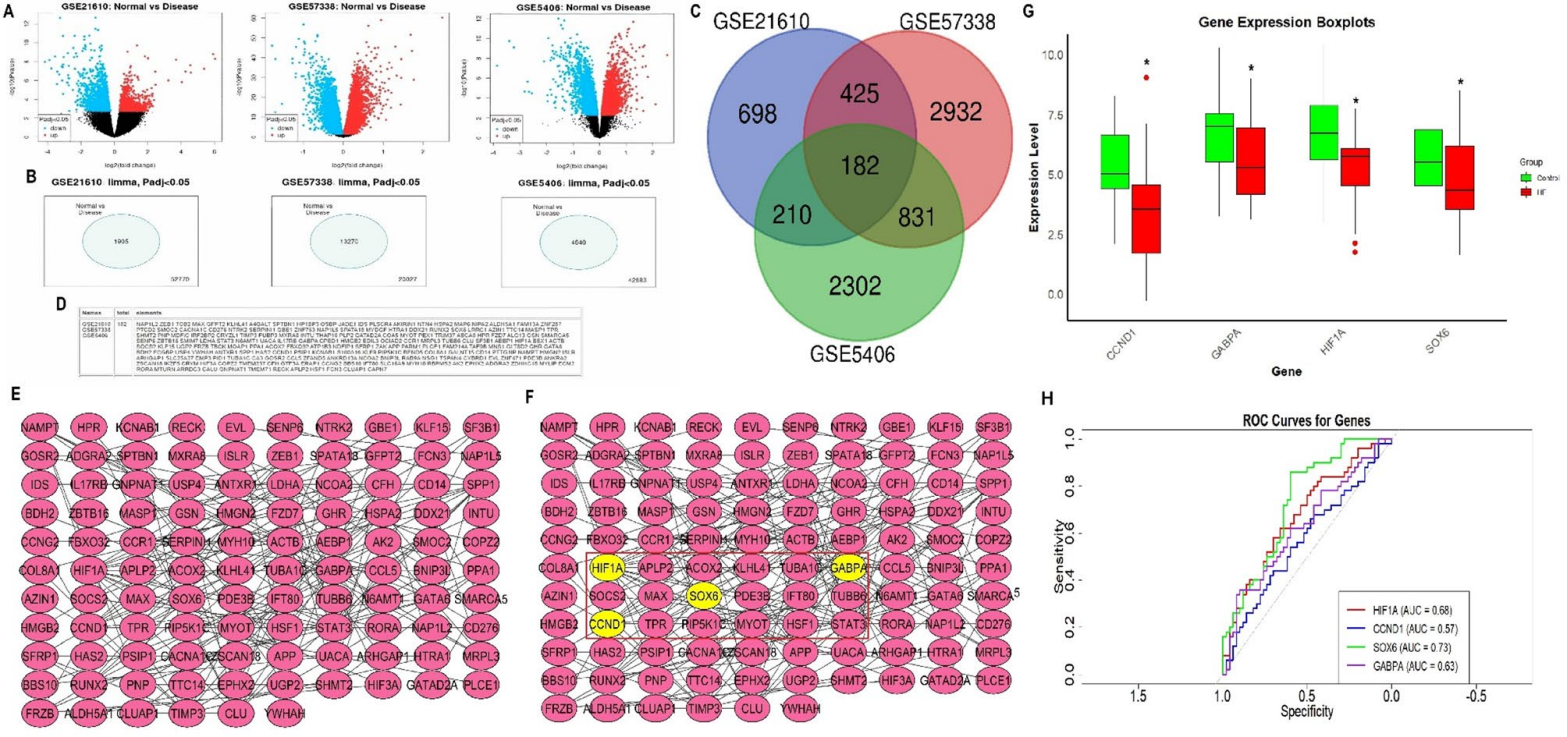
Identification of Differentially Expressed Genes (DEGs) and hub genes in HF (HF) patients. (**A-B**) Volcano and Venn plots displaying the most significantly upregulated and downregulated DEGs between HF and normal control samples from the GSE21610, GSE57338, and GSE5406 datasets. (**C**) Venn diagram showing the common DEGs identified across the three datasets. (**D**) PPI network of the common DEGs constructed using STRING database and analyzed with Cytoscape. (**E-F**) Identification of hub genes (CCND1, GABPA, HIF1A, and SOX6) using the CytoHubba application in Cytoscape based on their degree of centrality. (**G**) Expression analysis of hub genes in five HF and five normal control cell lines. (**H**) ROC curves generated for the identified hub genes. P*-value < 0.05

In total, three expression datasets of HF and normal control samples, including GSE21610, GSE57338, and GSE5406 were retrieved from the GEO database. These datasets were analyzed using limma package to identify DEGs between HF and normal control samples. The volcano and Venn plots in Fig. 1A-B provided a clear overview of the genes with the most significant changes in expression, thereby highlighting the DEGs associated with HF (Fig. 1A-B). To further refine our results, a Venn analysis of the significantly dysregulated DEGs was performed across all datasets. This analysis identified 182 common DEGs that are critical regulators of HF (Fig. 1C). Next, a PPI network of the common DEGs was constructed using the STRING database and analyzed with Cytoscape software. CytoHubba application of Cytoscape revealed CCND1, GABPA, HIF1A, and SOX6 as hub genes having high degrees of centrality (Fig. 1E-F). Expression analysis of the identified hub genes was conducted across five HF cell lines and five normal control cell lines. The results showed significant (*p*-value < 0.05) down-regulation of CCND1, GABPA, HIF1A, and SOX6 hub genes in HF cell lines relative to normal controls (Fig. 1G). Finally, to assess the diagnostic potential of the identified hub genes, ROC curves were generated using RT-qPCR data. The ROC analysis demonstrated that the identified hub genes, particularly HIF1A, CCND1, SOX6, and GABPA, exhibit strong discriminatory power between HF and normal control samples (Fig. 1H).

### Construction of risk development model and immune cell infiltration analysis

**Fig. 2:**
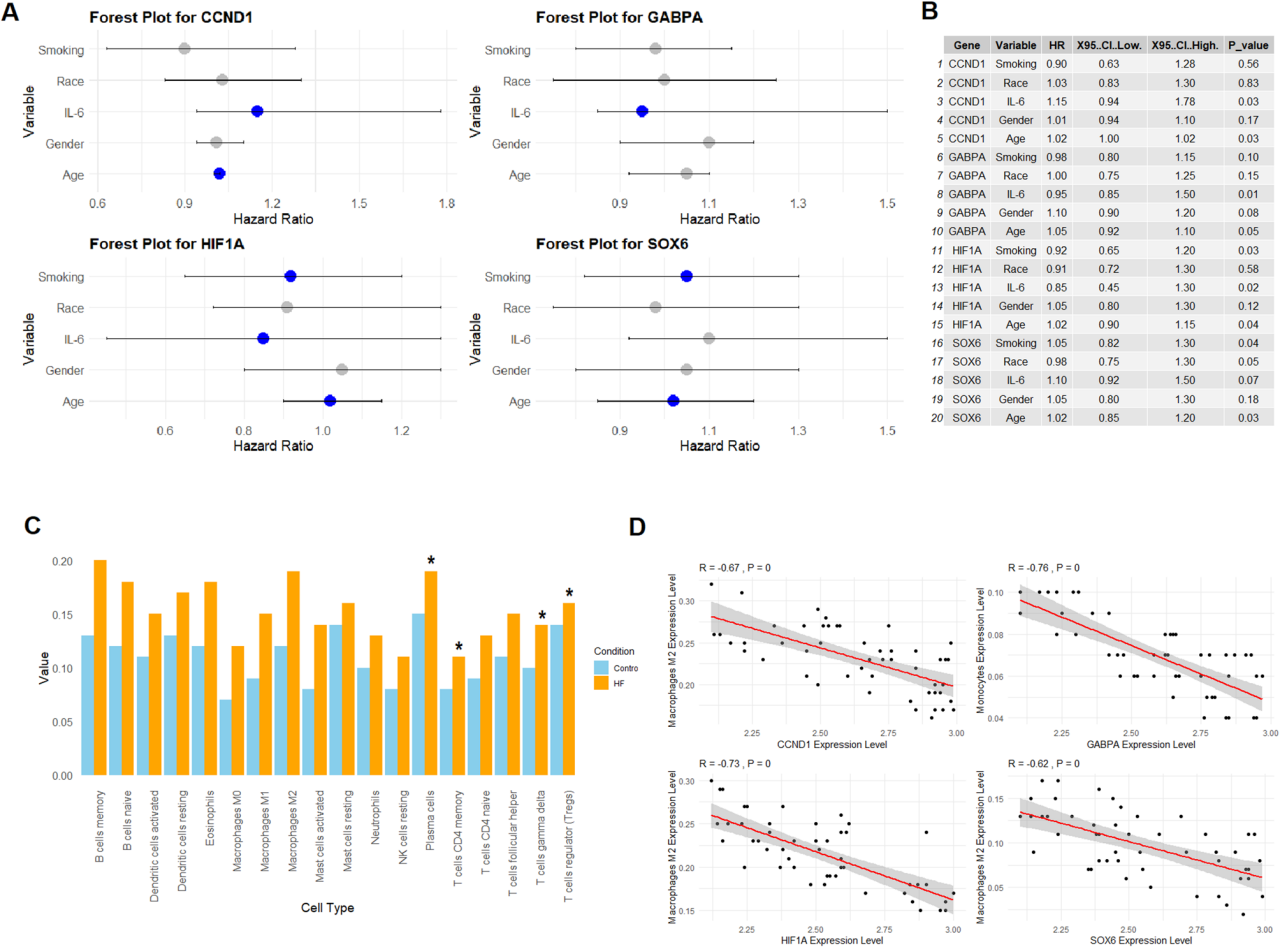
Risk model development and immune cell infiltration in HF patients. (**A-B**) LASSO regression analysis used to build a risk model based on the identified hub genes. (**C**) Immune cell infiltration analysis of the GSE21610 dataset using the CIBERSORT algorithm. (**D**) Pearson correlation analysis showing the immune cells most closely associated with the identified hub genes. P*-value < 0.05

In the next part of the study, we utilized LASSO regression analysis to build a risk model for HF patients based on the hub genes. LASSO regression was employed predict prognostic values of the hub genes across different clinical variables. Smoking, race, IL-6, gender, and age variables were evaluated for their influence on gene expression using univariate Cox regression analyses. For CCND1, the analysis suggests no significant associations between gene expression and smoking, race, or gender, with HR values close to 1 and *p*-values above 0.05, indicating that these variables may not be strong predictors of disease outcome in this context (Fig. 2A-B). However, a significant association was observed for IL-6, with a HR of 1.15 (95% CI: 0.94–1.78, *p* = 0.03), suggesting that increased levels of IL-6 may be associated with poorer prognosis in patients expressing CCND1 (Fig. 2A-B). Additionally, age emerged as another significant factor, with a HR of 1.02 (95% CI: 1.00–1.15, *p* = 0.03), implying that older age may contribute to worse outcomes in individuals with elevated CCND1 expression, possibly due to age-related alterations in cell cycle regulation (Fig. 2A-B). Similarly, for GABPA, the associations with smoking, race, and gender were not significant, as indicated by HRs close to 1 and non-significant *p*-values (*p* > 0.05) (Fig. 2A-B). However, the analysis highlighted a significant association with IL-6 (HR = 1.10, 95% CI: 0.80–1.50, *p* = 0.01), reinforcing the potential role of GABPA in inflammatory pathways, where higher IL-6 levels correlate with adverse disease outcomes (Fig. 2A-B). This suggests that GABPA might be a key regulator of inflammation in the tumor microenvironment. The results for HIF1A also showed some intriguing patterns. While no significant associations were observed with race or gender, the variable IL-6 exhibited a statistically significant HR of 1.05 (95% CI: 0.90–1.20, *p* = 0.04) (Fig. 2A-B), supporting the hypothesis that HIF1A is involved in the hypoxic stress response and may influence disease progression in the context of inflammation. Finally, SOX6 demonstrated an interesting relationship with IL-6, as the HR for IL-6 was 1.10 (95% CI: 0.85–1.50, *p* = 0.03) (Fig. 2A-B), suggesting that SOX6 expression may be associated with poorer prognosis in the presence of increased inflammation. Although age and smoking did not show significant correlations (Fig. 2A-B), the IL-6-related findings further emphasize the importance of inflammation as a driver of disease progression, particularly in the context of SOX6 expression. Additionally, immune cell infiltration in the GSE21610 dataset was analyzed using the CIBERSORT algorithm. This algorithm was used to quantify the relative proportions of various immune cell types in the heart tissue of HF patients. Given the known association between immune cells and HF, this analysis provided important insights into how immune cell profiles may contribute to the disease. The results from this analysis (Fig. 2C) demonstrated significant (*p*-value < 0.05) differences in the immune cell expression between HF patients and controls. Specifically, plasma cells, T cells CD4 memory activated, T cells regulatory (Tregs), and T cells gamma delta were significantly (*p*-value < 0.05) higher in the left ventricle of HF patients compared to controls (Fig. 2C). These findings suggest that these immune cells play a role in the inflammatory processes and tissue remodeling associated with HF. To further explore the relationship between immune cells and hub genes, Pearson correlation analysis was performed (Fig. 2D). This analysis revealed the macrophages and monocytes immune cells were most closely associated with the hub genes (Fig. 2D).

### PPI network construction gene enrichment analysis of hub genes

**Fig. 3:**
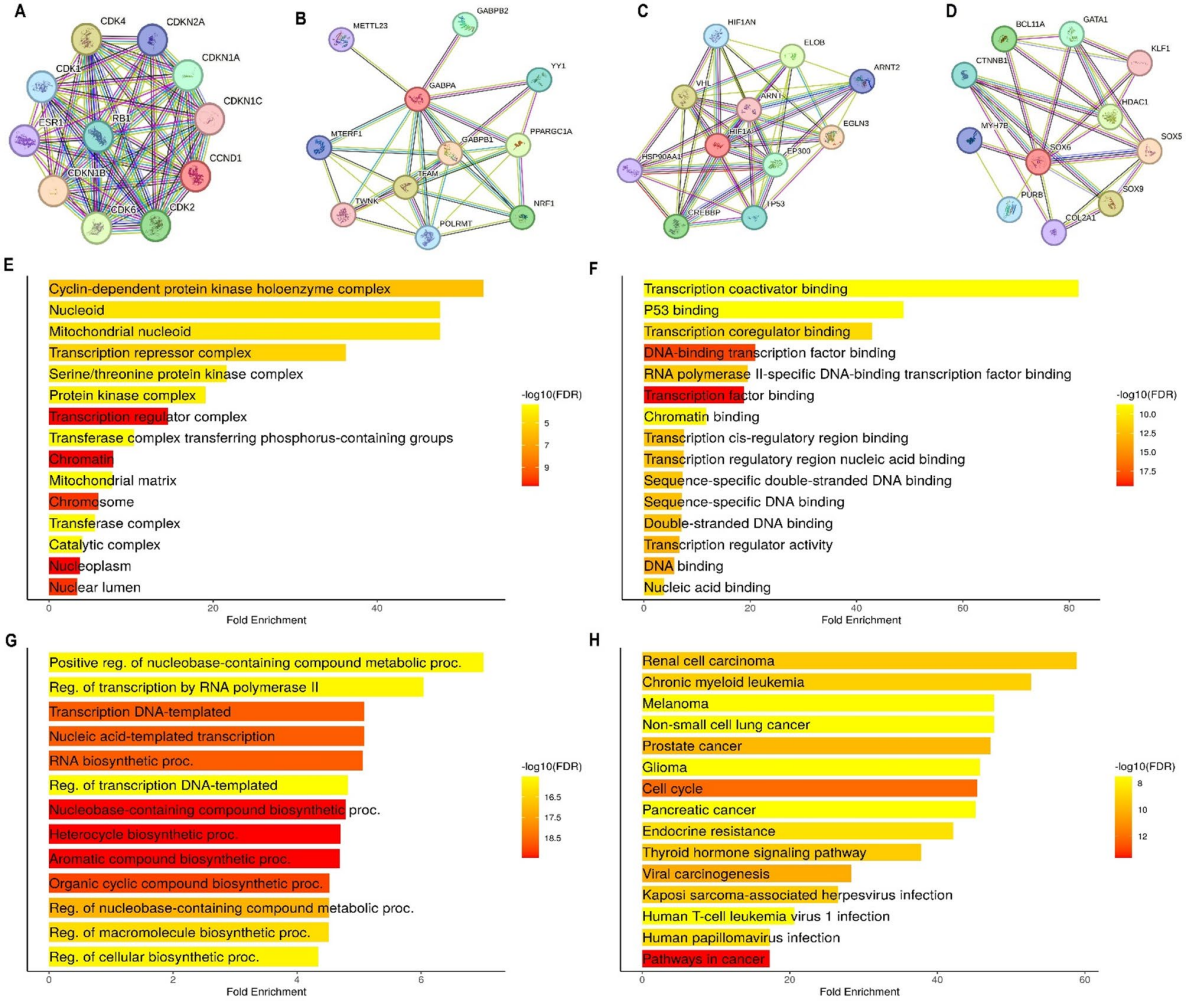
PPI network construction and gene enrichment analysis of hub genes. (**A-D**) PPI networks for the hub genes identified via the STRING database. (**E**) Cellular component analysis (**F**) Molecular function analysis. (**G**) Biological process analysis. (**H**) KEGG pathway analysis. Enrichment threshold of *P*-value < 0.05 was used

In this part of the study, we analyzed the interactions and functional enrichment of hub genes. The PPI networks for the hub genes identified via the STRING database are shown in Fig. 3A-D. The networks demonstrate that these hub genes are central to several other binding partners (Fig. 3A-D). Next, the gene enrichment analysis of hub gene binding partners was performed using DAVID to explore associated cellular components, molecular functions, biological processes, and KEGG pathways. Enriched cellular components included the cyclin-dependent protein kinase holoenzyme complex, nucleoid, mitochondrial matrix, and chromatin (Fig. 3E). Key molecular functions involved transcription coactivator binding, P53 binding, and DNA-binding transcription factor binding (Fig. 3F). Biological processes were mainly related to transcriptional regulation and nucleic acid metabolism (Fig. 3F). KEGG pathways such as renal cell carcinoma, chronic myeloid leukemia, and endocrine resistance were enriched, indicating potential roles of hub genes in cancer signaling and possible therapeutic relevance to HF (Fig. 3G).

### Expression validation analysis of hub genes

**Fig. 4:**
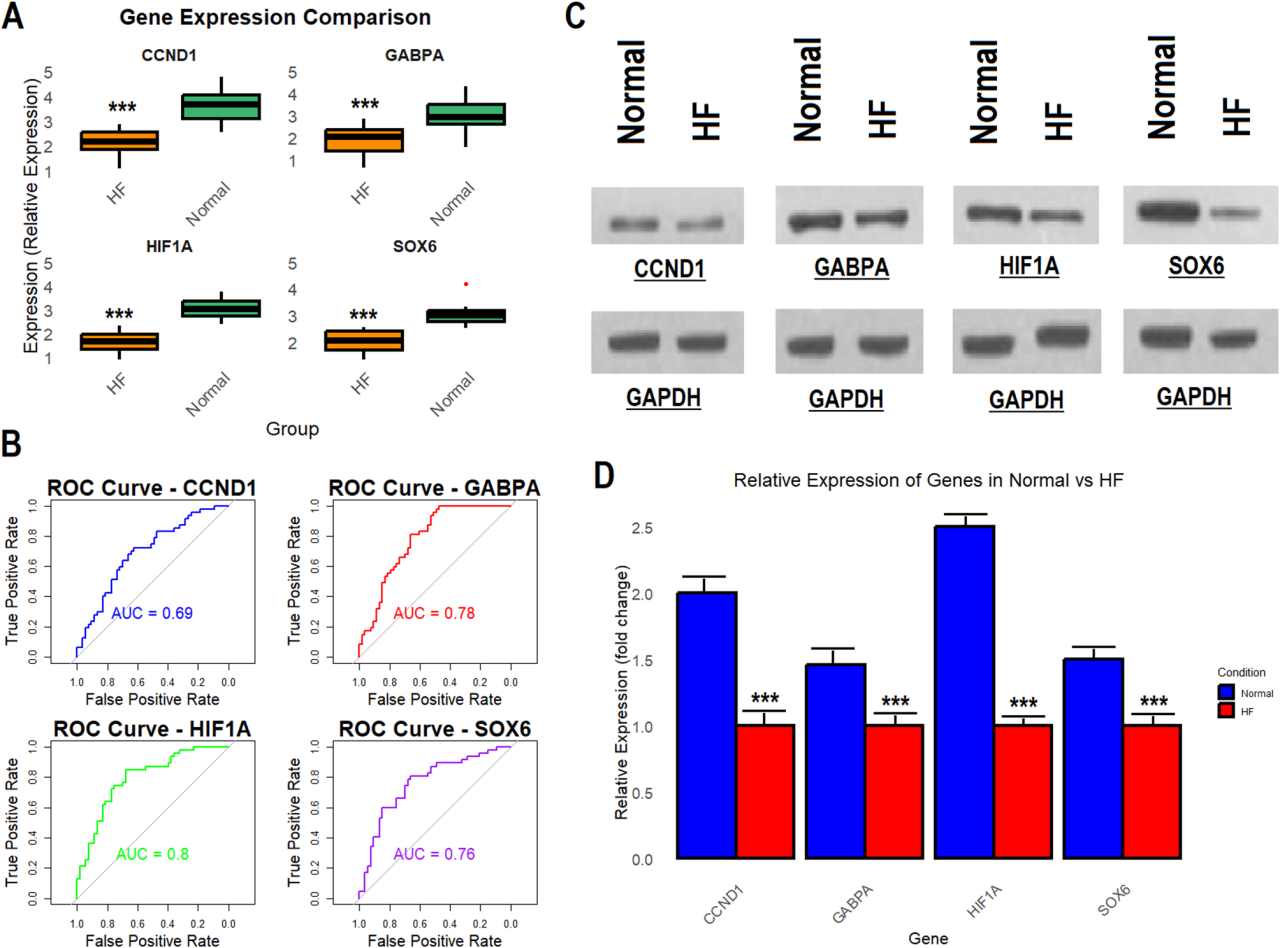
Expression validation of hub genes at the mRNA and protein levels. (**A**) mRNA expression validation of hub genes CCND1, GABPA, HIF1A, and SOX6 using data from the GSE57345 dataset. (**B**) ROC curves for CCND1, GABPA, HIF1A, and SOX6. (**C-D**). Western blot analysis of CCND1, GABPA, HIF1A, and SOX6 in HF and normal control cell lines. P***-value < 0.001

Next, we validated the expression of the hub genes at both the mRNA and protein levels to further investigate their potential as biomarkers for HF. The mRNA expression of the hub genes CCND1, GABPA, HIF1A, and SOX6 was first validated using data from the GSE57345 dataset having 177 HF samples and 136 normal control samples. The results showed that the expression of CCND1, GABPA, HIF1A, and SOX6 was significantly (*p*-value < 0.0001) lower in the HF sample group compared to controls (Fig. 4A). To assess the diagnostic potential of the hub genes, we generated ROC curves for CCND1, GABPA, HIF1A, and SOX6. The ROC curves revealed that all four hub genes exhibited strong discriminatory power between HF and normal control samples, with HIF1A showing the highest AUC of 0.80, followed by GABPA (AUC = 0.78), SOX6 (AUC = 0.76), and CCND1 (AUC = 0.69) (Fig. 4B). These AUC values indicate that these genes can effectively differentiate HF patients from normal individuals, supporting their potential as diagnostic biomarkers for HF. Next, we validated the protein expression levels of the hub genes across 5 HF cell line samples and 5 normal control cell line samples using Western blot analysis. The protein expression results were consistent with the mRNA data, showing significantly (*p*-value < 0.05) lower expression levels of CCND1, GABPA, HIF1A, and SOX6 in the HF cell lines compared to the normal controls (Fig. 4C-D and Supplementary data Fig. 1).

### miRNA-mRNA network construction and analysis

**Fig. 5:**
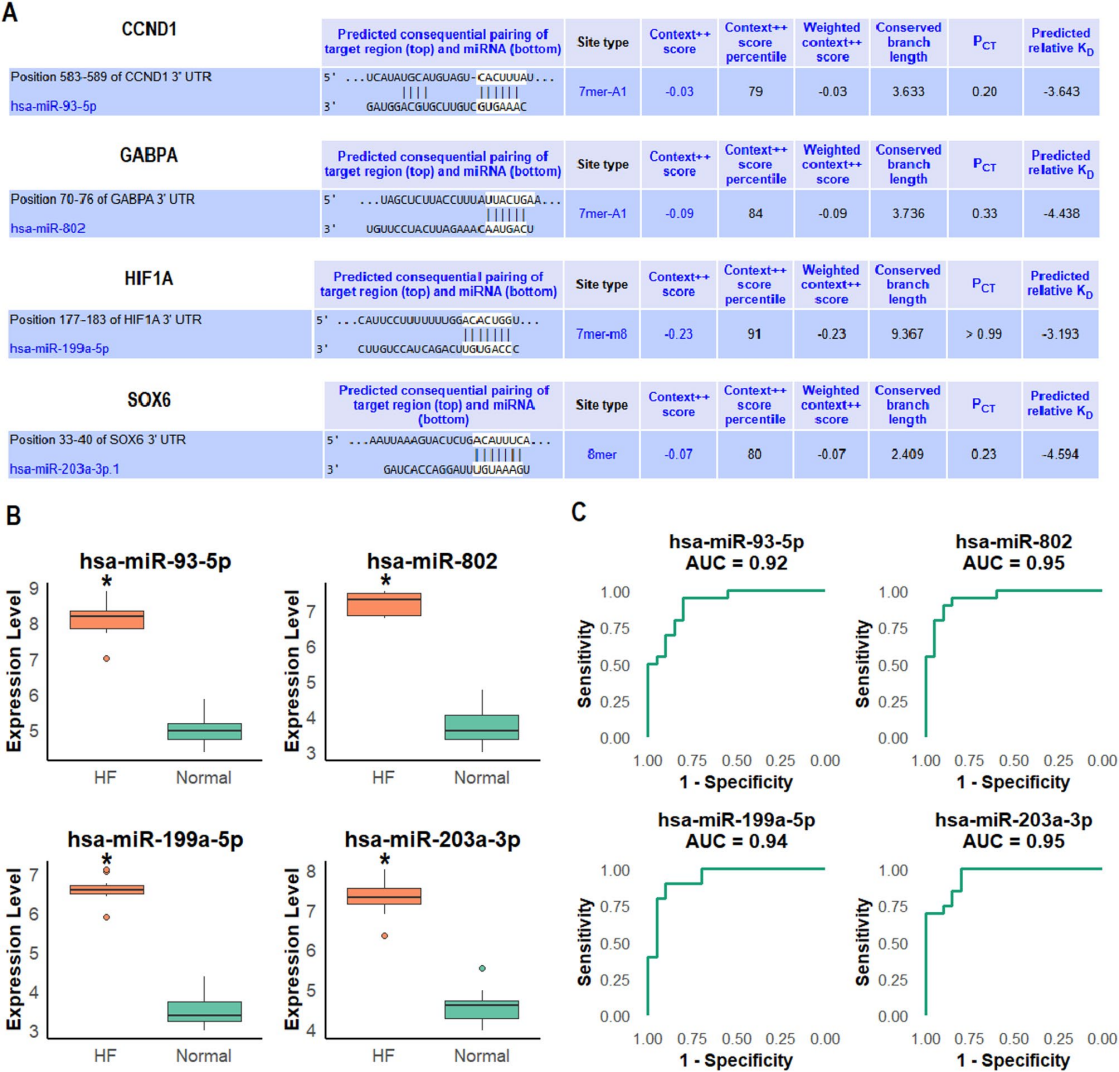
miRNA-mRNA network construction and analysis. (**A**) miRNAs predicted to target the 3’ untranslated regions (UTRs) of the hub genes CCND1, GABPA, HIF1A, and SOX6, using the Human TargetScan database. (**B**) Expression analysis of the predicted miRNAs hsa-miR-93-5p, hsa-miR-802, hsa-miR-199a-5p, and hsa-miR-203a-3p in five HF and five normal control cell lines using RT-qPCR. (**C**) ROC curves generated for each of the predicted miRNAs, demonstrating strong discriminatory power for hsa-miR-93-5p, hsa-miR-802, hsa-miR-199a-5p, and hsa-miR-203a-3p. P*-value < 0.05

We investigated the potential regulatory role of miRNAs in controlling the expression of hub genes in HF. Using the Human TargetScan database, we predicted the miRNAs that may target the 3’ UTRs of the hub genes CCND1, GABPA, HIF1A, and SOX6. The predicted miRNAs and their corresponding binding sites on the target gene 3’ UTRs are summarized in Fig. 5A. For each gene, we identified the position of the target site and the corresponding miRNA. The context scores, which indicate the strength of miRNA binding to the target gene, as well as the predicted relative binding affinities, are also provided. For example, hsa-miR-93-5p was predicted to target CCND1 with a context score of -0.03 and predicted relative binding affinity (Kd = -3.643) (Fig. 5A). Similarly, hsa-miR-802, hsa-miR-199a-5p, and hsa-miR-203a-3p were predicted to target GABPA, HIF1A, and SOX6, respectively (Fig. 5A). RT-qPCR analysis revealed that hsa-miR-93-5p, hsa-miR-802, hsa-miR-199a-5p, and hsa-miR-203a-3p were significantly upregulated in HF cell lines compared to controls (*p* < 0.05, Fig. 5B). ROC curve analysis demonstrated strong diagnostic potential for all four miRNAs, with AUC values ranging from 0.94 to 0.95, indicating their effectiveness as potential biomarkers for HF (Fig. 5C).

### Screening of potential therapeutic drugs

**Fig. 6:**
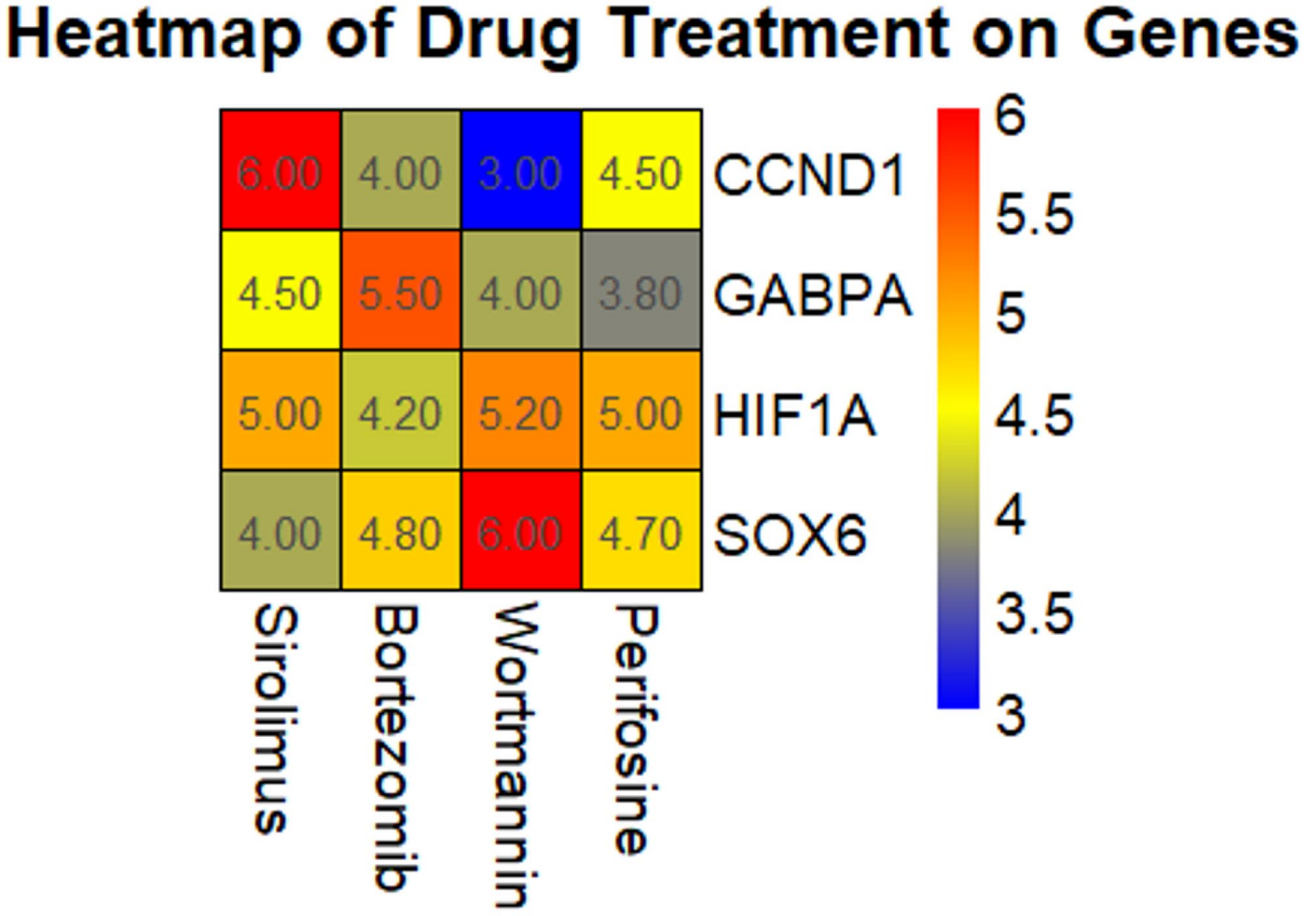
Screening of potential therapeutic drugs using the cMap database. A Heatmap displaying the relationship between the expression levels of the hub genes CCND1, GABPA, HIF1A, and SOX6 and different drug treatments, including Sirolimus, Bortezomib, Wortmannin, and Perifosine. *P*-value < 0.0001

In this part of the study, we used the cMap database to identify small molecule compounds that are negatively related to the hub genes (*p* < 0.0001 and mean < 0.4). The heatmap displays the relationship between the expression levels of the hub genes (CCND1, GABPA, HIF1A, and SOX6) and different drug treatments, including Sirolimus, Bortezomib, Wortmannin, and Perifosine (Fig. 6). The results demonstrate that Sirolimus, Bortezomib, and Wortmannin have varying effects on the expression of the hub genes. Specifically, Sirolimus was associated with higher expression levels of CCND1 and GABPA (Fig. 6), while it showed a more modest effect on HIF1A and SOX6 (Fig. 6). Bortezomib led to increased expression of HIF1A and SOX6, suggesting its potential role in regulating these genes (Fig. 6). Wortmannin, on the other hand, was associated with higher expression of CCND1 and GABPA, but lower expression of SOX6, indicating a selective effect on certain hub genes (Fig. 6). The drug Perifosine appeared to have the most pronounced effects, with a notable increase in the expression of SOX6 while maintaining lower expression levels of CCND1, GABPA, and HIF1A (Fig. 6).

### Induction of CCND1, HIF1A overexpression and analysis of cell proliferation, colony formation, and wound healing abilities of AC16 and SEKHEP1 cells

**Fig. 7:**
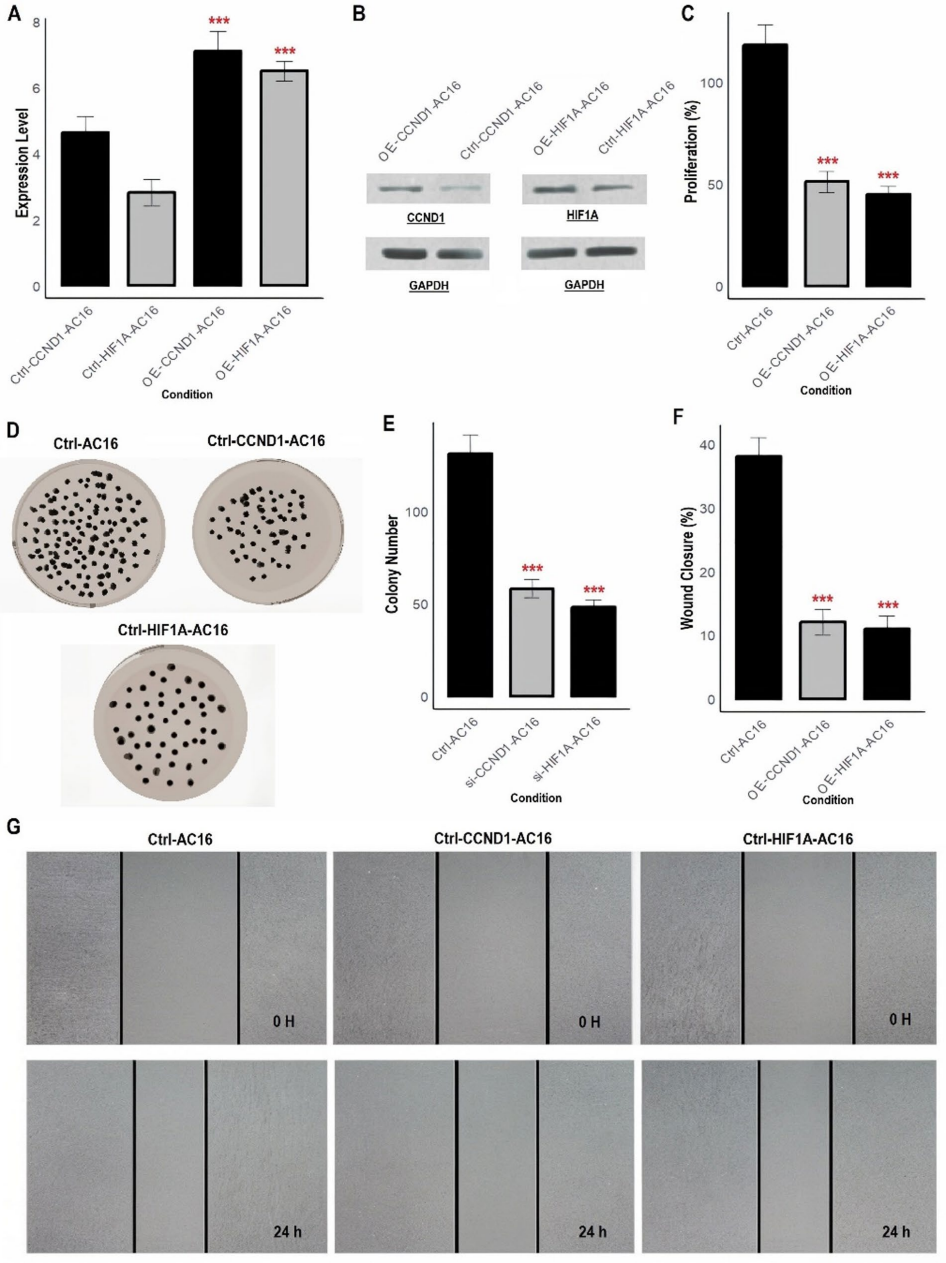
Induction of CCND1 and HIF1A overexpression and analysis of cellular functions in AC16 cells. (**A**) mRNA expression levels of CCND1 and HIF1A in the overexpressing AC16 were significantly elevated compared to the control cells. (**B**) Western blot analysis confirming the significant upregulation of CCND1 and HIF1A at the protein level in the overexpressing cell lines, in line with the mRNA expression results. (**C**) Proliferation assay showing a significant reduction in cell proliferation in OE-AC16 cells compared to control cells. (**D-E**) Colony formation assay demonstrating that overexpression of CCND1 and HIF1A resulted in significantly fewer colonies formed in OE-AC16 cells. (**F-G**) Wound closure assay showing that overexpression of CCND1 and HIF1A significantly delayed wound closure in OE-AC16 cells. P***-value < 0.001

**Fig. 8:**
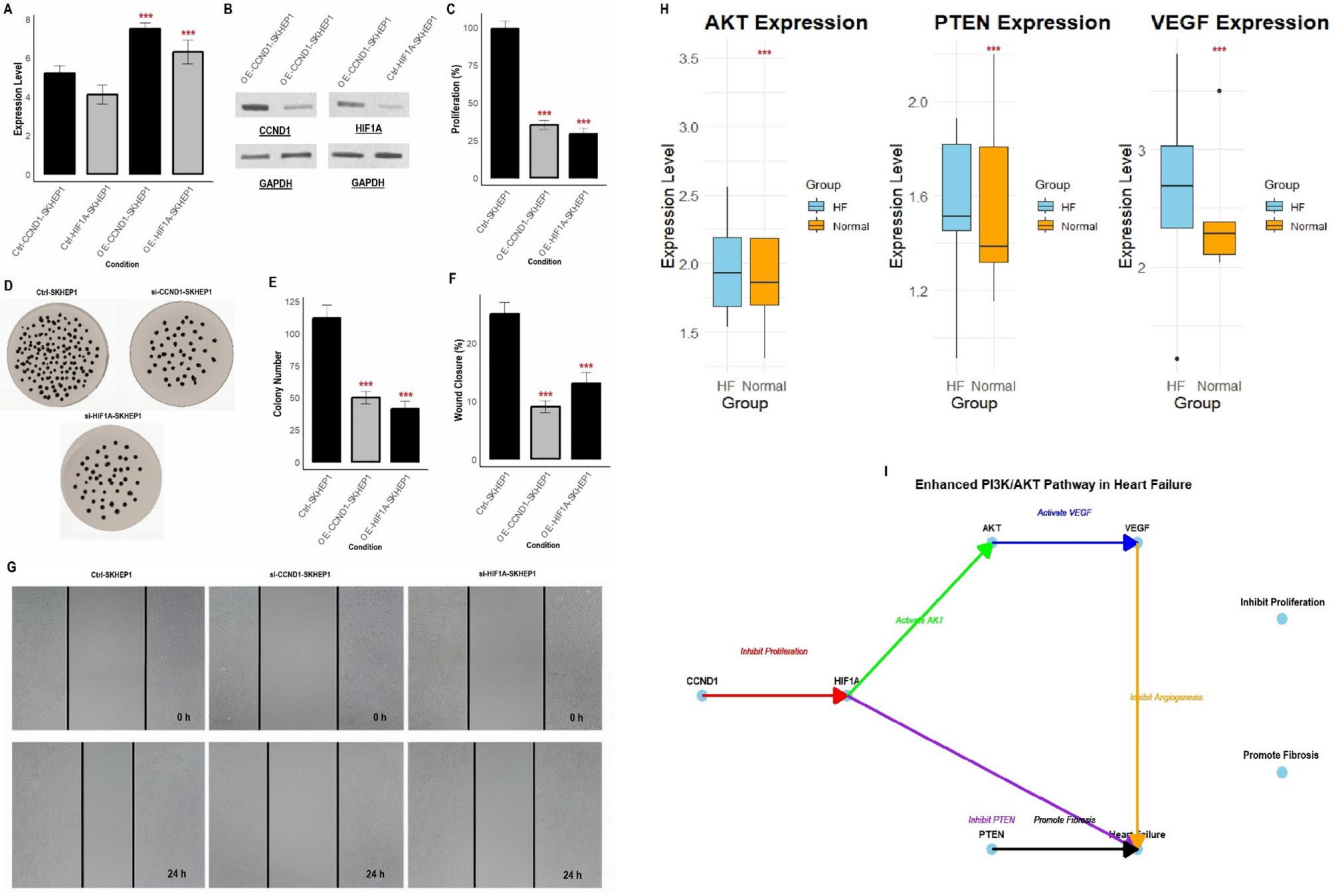
Induction of CCND1 and HIF1A overexpression and analysis of cellular functions in SEKHEP1 cells. (**A**) mRNA expression levels of CCND1 and HIF1A in the overexpressing SEKHEP1 were significantly elevated compared to the control cells. (**B**) Western blot analysis confirming the significant upregulation of CCND1 and HIF1A at the protein level in the overexpressing cell lines, in line with the mRNA expression results. (**C**) Proliferation assay showing a significant reduction in cell proliferation in OE- SEKHEP1 cells compared to control cells. (**D-E**) Colony formation assay demonstrating that overexpression of CCND1 and HIF1A resulted in significantly fewer colonies formed in OE- SEKHEP1 cells. (**F-G**) Wound closure assay showing that overexpression of CCND1 and HIF1A significantly delayed wound closure in OE- SEKHEP1 cells. (**H**) Expression analysis of AKT, PTEN, and VEGF in five HF and five normal control cell lines using RT-qPCR. (**I**) Cross-talk model of the PI3K/AKT pathway in HF, illustrating how CCND1 and HIF1A regulate the pathway by interacting with AKT, PTEN, and VEGF. P***-value < 0.001

The overexpression of CCND1 and HIF1A was induced using expression vectors in AC16 and SEKHEP1 cardiomyocyte cell lines to assess their functional impact on HF-related cellular processes. The mRNA expression levels of CCND1 and HIF1A in the overexpressing cell lines (OE-AC16 and OE-SEKHEP1) were significantly elevated compared to the control cells (Ctrl-AC16 and Ctrl-SEKHEP1), confirming successful gene induction at the transcriptional level (Figs. 7A and 8A). At the protein level, CCND1 and HIF1A were also significantly upregulated in the overexpressing cell lines, as confirmed by Western blot analysis. The increase in protein levels corroborates the mRNA expression findings and verifies the successful induction of both genes at the protein level (Figs. 7B and 8B, and Supplementary data Fig. 1). In the proliferation assay, the overexpression of CCND1 and HIF1A led to a significant reduction in cell proliferation compared to control cells (Figs. 7C and 8C). The colony formation assay revealed that overexpression of CCND1 and HIF1A resulted in significantly fewer colonies formed, indicating a reduction in clonogenic potential (Figs. 7D-E and 8D-E). This suggests that these genes may impair the growth and regeneration of cardiomyocytes, which could exacerbate the pathophysiology of HF. In the wound closure assay, the overexpression of CCND1 and HIF1A significantly impaired wound closure compared to control cells (Figs. 7F-G and 8F-G). This finding indicates that these genes inhibit cell migration, a crucial process for tissue repair and regeneration.

CCND1 and HIF1A are integral parts of the PI3K/AKT signaling pathway. To investigate the role of this pathway in HF, we analyzed the expression of some key genes involved in the PI3K/AKT pathway. In Fig. 8H, we analyzed the expression AKT, PTEN, and VEGF, across 5 HF and 5 normal control cell lines using RT-qPCR. The results showed that the expression of AKT, PTEN, and VEGF was significantly down-regulated in HF cells compared to controls (Fig. 8H). Finally, a cross-talk model of the PI3K/AKT pathway in HF was developed (Fig. 8I). The model illustrates how CCND1 and HIF1A regulate the PI3K/AKT pathway by interacting with AKT and VEGF, promoting fibrosis and inhibiting cell proliferation (Fig. 8I). The model highlights the complex interactions between these genes and their role in HF, where down-regulation of CCND1 and HIF1A may enhance the PI3K/AKT pathway, contributing to cardiac dysfunction.

## Discussion

Heart failure (HF) is a debilitating clinical syndrome characterized by the heart’s inability to pump sufficient blood to meet the body’s demands, resulting in fluid retention, shortness of breath, and fatigue [41, 49]. It affects millions of individuals worldwide, contributing to high morbidity, mortality, and healthcare costs [50, 51]. The disease progresses through various stages, from asymptomatic to overt HF, eventually leading to end-stage cardiac dysfunction [52]. Despite advances in pharmacological treatments, the prognosis for HF patients remains poor, with many therapies focusing on alleviating symptoms rather than addressing the underlying molecular mechanisms driving the disease [53, 54]. Therefore, understanding the molecular drivers of HF and identifying novel biomarkers for diagnosis and prognosis are critical for developing more effective treatments. While HF is known to involve complex molecular pathways, the precise mechanisms by which specific genes and signaling pathways contribute to disease progression remain incompletely understood. Previous studies have suggested the involvement of various genes and signaling pathways in HF [55–57], but comprehensive analyses integrating multiple datasets and considering the role of key hub genes within well-established signaling pathways, such as the PI3K/AKT pathway, are lacking. Moreover, the dysregulation of immune cell infiltration in HF and its impact on disease progression has not been extensively studied in conjunction with gene expression and pathway analysis. There is a clear need to identify key genes, investigate their roles in HF, and explore potential therapeutic strategies targeting these molecules.

Our study aimed to fill these gaps by identifying and characterizing key hub genes involved in HF using integrated bioinformatics and experimental approaches. We combined DEG analysis from multiple datasets, PPI network analysis, immune cell infiltration analysis, and gene enrichment analysis to explore the role of these genes in HF. By analyzing CCND1, GABPA, HIF1A, and SOX6, we sought to identify key regulators of disease progression and investigate the dysregulation of the PI3K/AKT pathway in the context of HF. This comprehensive approach allowed us to uncover novel biomarkers for HF diagnosis and prognosis and identify potential therapeutic targets for future interventions.

Several studies have previously investigated the roles of CCND1, GABPA, HIF1A, and SOX6 in various diseases, including cancer and cardiovascular diseases [58–61], but the specific roles of these genes in HF and their interaction within the context of the PI3K/AKT pathway have not been well-explored. While individual studies have shown that CCND1 and HIF1A are crucial players in cell proliferation and migration, particularly in cardiac hypertrophy and vascular remodeling [61, 62], the interaction of these genes within the PI3K/AKT pathway in HF is still not fully understood. CCND1 has been implicated in the regulation of the cell cycle and cardiac hypertrophy in HF [61], and HIF1A is known to be involved in angiogenesis and tissue remodeling under hypoxic conditions, both of which are essential processes in HF progression [62]. Our study confirms these findings and expands upon them by showing that dysregulation of CCND1 and HIF1A in HF cells leads to impaired cell proliferation and migration, exacerbating the pathological changes observed in the disease.

In addition, the role of GABPA, a transcription factor involved in mitochondrial function and oxidative stress response, has been previously studied in various diseases [63, 64], but its specific role in HF has not been as extensively investigated. In our study, GABPA was identified as a critical hub gene involved in HF, and we found that its downregulation in HF cells contributes to mitochondrial dysfunction and metabolic disturbances, which are common features in HF [64]. These findings align with previous research that suggests GABPA plays a significant role in regulating cell metabolism [65], but our study further emphasizes its importance in maintaining mitochondrial integrity in the context of HF.

Similarly, SOX6, a transcription factor involved in cardiac development and repair, has been shown to regulate cardiac remodeling and tissue regeneration [61, 66]. Our study adds to this understanding by demonstrating that SOX6 is downregulated in HF, impairing cell migration and tissue repair mechanisms. The loss of SOX6 expression in HF cells could contribute to the reduced regenerative capacity observed in the disease, supporting the idea that SOX6 plays a protective role in maintaining cardiac function and preventing adverse remodeling in HF.

One of the novel aspects of our study is the integration of immune cell infiltration analysis and the expression analysis of hub genes in HF. Previous studies have suggested that immune cells, particularly macrophages and monocytes, play a role in the inflammatory response and tissue remodeling in HF [67, 68]. Our study provides a more detailed analysis by correlating the infiltration of these immune cells with the expression of key hub genes, revealing a close association between immune activation and gene dysregulation in HF. This adds a new layer of complexity to the pathophysiology of HF, highlighting the importance of considering both genetic and immune factors in understanding the disease.

Furthermore, our study is unique in its use of miRNA-mRNA network construction to identify potential post-transcriptional regulation of hub genes. By predicting miRNAs that regulate CCND1, GABPA, HIF1A, and SOX6, we provided new insights into the role of miRNAs in HF. The strong discriminatory power of the identified miRNAs, particularly hsa-miR-93-5p, hsa-miR-802, hsa-miR-199a-5p, and hsa-miR-203a-3p, further supports their potential as diagnostic biomarkers for HF.

In addition to identifying potential biomarkers, our study also screened for small molecule compounds using the cMap database, offering a preliminary strategy to discover potential therapeutic agents that may modulate the expression of hub genes involved in HF. Compounds such as Sirolimus, Bortezomib, Wortmannin, and Perifosine were predicted to influence gene expression patterns. While these drugs show theoretical promise, their clinical translation requires rigorous validation, including assessment of in vivo efficacy, pharmacodynamics, and long-term safety profiles. Moreover, some of these agents are associated with known adverse effects or limited therapeutic windows, which may constrain their application in HF treatment. Further experimental and clinical investigations are essential to evaluate the feasibility and therapeutic relevance of these candidate compounds.

Furthermore, our exploration of the PI3K/AKT pathway and its dysregulation in HF, facilitated by CCND1 and HIF1A, represents an important advancement in understanding the molecular drivers of the disease. The development of a cross-talk model of the PI3K/AKT pathway [69, 70] in HF that incorporates CCND1 and HIF1A provides a fresh perspective on how these genes might work together to promote fibrosis, inhibit proliferation, and contribute to HF progression. This model lays the groundwork for potential therapeutic strategies targeting these key regulators.

Despite the comprehensive design and multi-omics integration employed in this study, a few limitations should be acknowledged. First, the sections “Expression validation analysis of hub genes” and “Overexpression of CCND1 and HIF1A in AC16 and SEKHEP1 cells” are limited by the lack of validation in in vivo models or human clinical cardiac tissue samples. The relatively small sample size and restricted biological diversity of the cell lines used may limit the generalizability of our findings. Secondly, while overexpression of CCND1 and HIF1A in cardiomyocyte cell lines revealed significant impacts on proliferation, migration, and colony formation, reverse experiments using gene knockdown or CRISPR-Cas9–mediated gene silencing were not conducted, which limits the mechanistic depth of our conclusions. Thirdly, in the “miRNA–mRNA network construction and analysis” section, only expression-level validations were performed after target prediction. Functional studies such as transfection of miRNA mimics or inhibitors and luciferase reporter assays for direct target validation were not included, which limits the causal interpretation of miRNA regulation. Fourthly, the CIBERSORT-based immune infiltration analysis infers immune cell composition from transcriptomic data without confirmatory assays such as flow cytometry or immunohistochemistry, which introduces a level of extrapolation that should be interpreted with caution. Fifth, while the enriched pathways such as Renal cell carcinoma and Melanoma were identified, the mechanistic links to HF remain unclear. These cancer-related pathways may involve shared signaling cascades, like PI3K/AKT, but their direct relevance to HF pathophysiology is speculative. Finally, while the drug prediction analysis from the cMap database identified candidate compounds, their effects were not validated experimentally in vitro or in vivo. Future studies incorporating animal models, patient-derived tissues, and functional immune and drug assays are essential to substantiate the translational potential of our findings and further elucidate the molecular mechanisms of HF.

## Conclusion

In conclusion, our study provides a comprehensive and integrated analysis of key hub genes and signaling pathways in HF. By identifying CCND1, HIF1A, GABPA, and SOX6 as critical regulators of disease progression and exploring their interaction with the PI3K/AKT pathway, we have made important strides in understanding the molecular mechanisms of HF. Our findings not only expand our understanding of the disease but also highlight potential biomarkers and therapeutic targets for future clinical interventions.

## Supplementary Information

Below is the link to the electronic supplementary material.

Supplementary Material 1

## Data Availability

The datasets analyzed during the current study are publicly available from the Gene Expression Omnibus (GEO) database at www.ncbi.nlm.nih.gov/geo/. Additional data supporting the findings of this study will be made available by the corresponding author upon reasonable request.

## Declarations

### Clinical trial number

This study does not involve a clinical trial. No human participants, human specimens, or animal specimens were used in this research.

## References

[1] Sulashvili N, Nimangre RR. Manifestation of some aspects of cardiovascular diseases, implications, pharmacotherapeutic strategies, effects, impacts and potential hazards in general. Junior Researchers. 2025;3(1):1–27.

[2] Parizad R, et al. Emerging risk factors for heart failure in younger populations: A growing public health concern. World J Cardiol. 2025;17(4):104717. 40308622 10.4330/wjc.v17.i4.104717 PMC12038706

[3] Pei W, et al. Multitargeted Immunomodulatory therapy for viral myocarditis by engineered extracellular vesicles. ACS Nano. 2024;18(4):2782–99. 38232382 10.1021/acsnano.3c05847

[4] Palaparthi EC et al. Emerging therapeutic strategies for heart failure: A comprehensive review of novel Pharmacological and molecular targets. Cureus, 2025. 17(4). 10.7759/cureus.81573 PMC12045464 40313442

[5] Li L, et al. Nanozyme-enhanced tyramine signal amplification probe for preamplification-free myocarditis-related MiRNAs detection. Chem Eng J. 2025;503:158093.

[6] Triposkiadis F, et al. Pathogenesis of chronic heart failure: cardiovascular aging, risk factors, comorbidities, and disease modifiers. Heart Fail Rev. 2022;27(1):337–44. 32524327 10.1007/s10741-020-09987-z

[7] Wei S, et al. Involvement of ROS/NLRP3 inflammasome signaling pathway in doxorubicin-induced cardiotoxicity. Cardiovasc Toxicol. 2020;20(5):507–19. 32607760 10.1007/s12012-020-09576-4

[8] Zhou Y, et al. Dermatophagoides Pteronyssinus allergen der p 22: cloning, expression, IgE-binding in asthmatic children, and immunogenicity. Pediatr Allergy Immunol. 2022;33(8):e13835. 36003049 10.1111/pai.13835

[9] Zhang Y, et al. M2 macrophage exosome-derived LncRNA AK083884 protects mice from CVB3-induced viral myocarditis through regulating PKM2/HIF-1α axis mediated metabolic reprogramming of macrophages. Redox Biol. 2023;69:103016. 38160539 10.1016/j.redox.2023.103016 PMC10792748

[10] Tepetes N-I, et al. Transition to advanced heart failure: from identification to improving prognosis. J Cardiovasc Dev Disease. 2025;12(3):104. 40137102 10.3390/jcdd12030104 PMC11943400

[11] Falco L, et al. Beyond medical Therapy—An update on heart failure devices. J Cardiovasc Dev Disease. 2024;11(7):187. 39057611 10.3390/jcdd11070187 PMC11277415

[12] Khan MS, et al. Global epidemiology of heart failure. Nat Reviews Cardiol. 2024;21(10):717–34. 10.1038/s41569-024-01046-6 38926611

[13] Roger VL. Epidemiology of heart failure: a contemporary perspective. Circul Res. 2021;128(10):1421–34. 10.1161/CIRCRESAHA.121.318172 33983838

[14] Lian W, et al. Celastrol improves isoproterenol-induced heart failure by reducing inflammation, apoptosis and oxidative stress. Int J Pharmacol. 2023;19(1):89–99.

[15] Wang W-z, et al. A novel small-molecule PCSK9 inhibitor E28362 ameliorates hyperlipidemia and atherosclerosis. Acta Pharmacol Sin. 2024;45(10):2119–33. 38811775 10.1038/s41401-024-01305-9 PMC11420243

[16] Fang Z, et al. Systemic aging fuels heart failure: molecular mechanisms and therapeutic avenues. ESC Heart Fail. 2025;12(2):1059–80. 39034866 10.1002/ehf2.14947 PMC11911610

[17] Fayyaz AU, et al. Pathophysiological insights into HFpEF from studies of human cardiac tissue. Nat Reviews Cardiol. 2025;22(2):90–104. 10.1038/s41569-024-01067-1 PMC11750620 39198624

[18] Ghazal R, et al. Cardiac fibrosis in the Multi-Omics era: implications for heart failure. Circul Res. 2025;136(7):773–802. 10.1161/CIRCRESAHA.124.325402 PMC11949229 40146800

[19] Casamassimi A, et al. Transcriptome profiling in human diseases: new advances and perspectives. Int J Mol Sci. 2017;18(8):1652. 28758927 10.3390/ijms18081652 PMC5578042

[20] Raghow R. An ‘omics’ perspective on cardiomyopathies and heart failure. Trends Mol Med. 2016;22(9):813–27. 27499035 10.1016/j.molmed.2016.07.007

[21] Cao L, et al. Identification of Co-diagnostic genes for heart failure and hepatocellular carcinoma through WGCNA and machine learning algorithms. Mol Biotechnol. 2024;66(5):1229–45. 38236461 10.1007/s12033-023-01025-1

[22] Ranganathan Y, et al. Understanding integrative approach of translational bioinformatics on cardiovascular disease: myocardial ischemia. Egypt J Med Hum Genet. 2024;25(1):153.

[23] Charles S, Natarajan J. Integrated regulatory network based on lncRNA-miRNA-mRNA-TF reveals key genes and sub-networks associated with dilated cardiomyopathy. Comput Biol Chem. 2021;92:107500. 33940530 10.1016/j.compbiolchem.2021.107500

[24] Khairnar M, Marda S. Integrative Multi-Omics in precision medicine: from molecular interconnectivity to Single-Cell resolution. Int J Sci R Tech, 2025. 2(7).

[25] Deng J, et al. The Janus face of mitophagy in myocardial ischemia/reperfusion injury and recovery. Volume 173. Biomedicine & Pharmacotherapy; 2024. p. 116337. 10.1016/j.biopha.2024.116337 38422659

[26] Qian F-C, et al. SEanalysis 2.0: a comprehensive super-enhancer regulatory network analysis tool for human and mouse. Nucleic Acids Res. 2023;51(W1):W520–7. 37194711 10.1093/nar/gkad408 PMC10320134

[27] Zhou M, et al. Comprehensive bioinformatics analysis of hub genes in ischemic heart failure and atrial fibrillation. Front Cardiovasc Med. 2025;12:1499065. 40066352 10.3389/fcvm.2025.1499065 PMC11891352

[28] Yuan Hj, et al. Identification of hub genes for the diagnosis associated with heart failure using multiple cell death patterns. ESC Heart Failure; 2025. 10.1002/ehf2.15299 PMC12287814 40205951

[29] Peng H, et al. Identification of Metabolism-Related hub genes in heart failure via comprehensive transcriptome analysis. Genes. 2025;16(3):305. 40149456 10.3390/genes16030305 PMC11941980

[30] Wang J-H, et al. CXCR6 deficiency attenuates pressure overload-induced monocytes migration and cardiac fibrosis through downregulating TNF-α-dependent MMP9 pathway. Int J Clin Exp Pathol. 2014;7(10):6514. 25400729 PMC4230124

[31] Gogiraju R, Bochenek ML, Schäfer K. Angiogenic endothelial cell signaling in cardiac hypertrophy and heart failure. Front Cardiovasc Med. 2019;6:20. 30895179 10.3389/fcvm.2019.00020 PMC6415587

[32] Zhang Z, Wang C. Exploring key genes and pathways of cardiac hypertrophy based on bioinformatics. Dis Markers. 2022;2022(1):2081590. 36046382 10.1155/2022/2081590 PMC9423995

[33] Li W-Q, et al. Calcitonin gene-related peptide inhibits the cardiac fibroblasts senescence in cardiac fibrosis via up-regulating Klotho expression. Eur J Pharmacol. 2019;843:96–103. 30352200 10.1016/j.ejphar.2018.10.023

[34] Barrett T, et al. NCBI GEO: mining millions of expression profiles—database and tools. Nucleic Acids Res. 2005;33(suppl1):D562–6. 15608262 10.1093/nar/gki022 PMC539976

[35] Ritchie ME, et al. Limma powers differential expression analyses for RNA-sequencing and microarray studies. Nucleic Acids Res. 2015;43(7):e47–47. 25605792 10.1093/nar/gkv007 PMC4402510

[36] Li S, et al. Modified lentiviral globin gene therapy for pediatric β0/β0 transfusion-dependent β-thalassemia: a single-center, single-arm pilot trial. Cell Stem Cell. 2024;31(7):961–73. e8. 38759653 10.1016/j.stem.2024.04.021

[37] Mering Cv, et al. STRING: a database of predicted functional associations between proteins. Nucleic Acids Res. 2003;31(1):258–61. 12519996 10.1093/nar/gkg034 PMC165481

[38] Hameed Y. Decoding the significant diagnostic and prognostic importance of maternal embryonic leucine zipper kinase in human cancers through deep integrative analyses. J Cancer Res Ther. 2023;19(7):1852–64. 38376289 10.4103/jcrt.jcrt_1902_21

[39] Saito R, et al. A travel guide to cytoscape plugins. Nat Methods. 2012;9(11):1069–76. 23132118 10.1038/nmeth.2212 PMC3649846

[40] Jiang F, et al. Key wound healing genes as diagnostic biomarkers and therapeutic targets in uterine corpus endometrial carcinoma: an integrated in Silico and in vitro study. Hereditas. 2025;162(1):5. 39833941 10.1186/s41065-025-00369-9 PMC11748876

[41] Zhang G, et al. TRAPT: a multi-stage fused deep learning framework for predicting transcriptional regulators based on large-scale epigenomic data. Nat Commun. 2025;16(1):3611. 40240358 10.1038/s41467-025-58921-0 PMC12003887

[42] Kukreja SL, Löfberg J, Brenner MJ. A least absolute shrinkage and selection operator (LASSO) for nonlinear system identification. IFAC Proc Volumes. 2006;39(1):814–9.

[43] Chen B et al. Profiling tumor infiltrating immune cells with CIBERSORT. Cancer Syst Biology: Methods Protocols, 2018: pp. 243–59. 10.1007/978-1-4939-7493-1_12 PMC5895181 29344893

[44] Hu H, et al. CDCA8, a mitosis-related gene, as a prospective pan-cancer biomarker: implications for survival prognosis and oncogenic immunology. Am J Transl Res. 2024;16(2):432–45. 38463578 10.62347/WSEF7878 PMC10918119

[45] Dennis G, et al. DAVID: database for annotation, visualization, and integrated discovery. Genome Biol. 2003;4:1–11.12734009

[46] Abdel-Maksoud MA, et al. Unlocking the diagnostic, prognostic roles, and immune implications of BAX gene expression in pan-cancer analysis. Am J Transl Res. 2024;16(1):63–74. 38322551 10.62347/TWOY1681 PMC10839381

[47] Singh NK. MiRNAs target databases: developmental methods and target identification techniques with functional annotations. Cell Mol Life Sci. 2017;74:2239–61. 28204845 10.1007/s00018-017-2469-1 PMC11107700

[48] Yang K, et al. CMAP: complement map database. Bioinformatics. 2013;29(14):1832–3. 23661693 10.1093/bioinformatics/btt269 PMC3702248

[49] Sugiura T, et al. From injury to repair: the therapeutic potential of induced pluripotent stem cells in heart failure. Regenerative Med Rep. 2025;2(1):22–30.

[50] Agarwal M, Pakhare SN. Emerging therapeutic strategies in heart failure management: A narrative review of current evidence and future directions. IJCS. 2024;6(1):32–8.

[51] Contractor H, Singh K, von Schwarz E. Cardiomyopathy Subclinical Heart Fail. 2024.

[52] Akunne OZ, Anulugwo OE. Emerging therapies targeting cardiovascular risk factors to prevent or delay the onset of heart failure. Am J Pharmacotherapy Pharm Sci, 2024. 3.

[53] Beghini A, et al. 2024 update in heart failure. ESC Heart Fail. 2025;12(1):8–42. 38806171 10.1002/ehf2.14857 PMC11769673

[54] Kitai T et al. JCS/JHFS 2025 guideline on diagnosis and treatment of heart failure. Circ J, 2025: p. CJ–25. 10.1253/circj.CJ-25-0002 40159241

[55] Fujita T, Ishikawa Y. Apoptosis in heart Failure–The role of the β-Adrenergic Receptor-Mediated signaling pathway and p53-Mediated signaling pathway in the apoptosis of Cardiomyocytes–. Circ J. 2011;75(8):1811–8. 21747195 10.1253/circj.cj-11-0025

[56] Ghiasi M. Investigating the NF-κB signaling pathway in heart failure: exploring potential therapeutic approaches. Heliyon, 2024. 10(23). 10.1016/j.heliyon.2024.e40812 PMC11664283 39717608

[57] Long-yan L et al. Research progress of related signal pathways in the prevention and treatment of heart failure with traditional Chinese medicine. J Hainan Med Univ, 2023. 29(12).

[58] Dehghani K, et al. CCND1 overexpression in idiopathic dilated cardiomyopathy: a promising biomarker? Genes. 2023;14(6):1243. 37372424 10.3390/genes14061243 PMC10298313

[59] Zhang S, et al. GABPA predicts prognosis and inhibits metastasis of hepatocellular carcinoma. BMC Cancer. 2017;17:1–12. 28549418 10.1186/s12885-017-3373-7 PMC5446731

[60] Semenza GL. Hypoxia-inducible factors: roles in cardiovascular disease progression, prevention, and treatment. Cardiovascular Res. 2023;119(2):371–80. 10.1093/cvr/cvac089 35687650

[61] Saleem M, Barturen-Larrea P, Gomez JA. Emerging roles of Sox6 in the renal and cardiovascular system. Physiological Rep. 2020;8(22):e14604. 10.14814/phy2.14604 PMC7683808 33230925

[62] Sato T, Takeda N. The roles of HIF-1α signaling in cardiovascular diseases. J Cardiol. 2023;81(2):202–8. 36127212 10.1016/j.jjcc.2022.09.002

[63] Zhou C et al. The transcription factor GABPA is a master regulator of Naive pluripotency. Nat Cell Biol, 2025: pp. 1–11. 10.1038/s41556-024-01554-0 PMC11735382 39747581

[64] Goto S, et al. Identification of GA-binding protein transcription factor alpha subunit (GABPA) as a novel bookmarking factor. Int J Mol Sci. 2019;20(5):1093. 30836589 10.3390/ijms20051093 PMC6429373

[65] Yang Z-F, Mott S, Rosmarin AG. The Ets transcription factor GABP is required for cell-cycle progression. Nat Cell Biol. 2007;9(3):339–46. 17277770 10.1038/ncb1548

[66] Saleem M, et al. Sox6, a potential target for MicroRNAs in cardiometabolic disease. Curr Hypertens Rep. 2022;24(5):145–56. 35124768 10.1007/s11906-022-01175-8 PMC12965638

[67] Shahid F, Lip GY, Shantsila E. Role of monocytes in heart failure and atrial fibrillation. J Am Heart Association. 2018;7(3):e007849. 10.1161/JAHA.117.007849 PMC5850261 29419389

[68] Hulsmans M, Sam F, Nahrendorf M. Monocyte and macrophage contributions to cardiac remodeling. J Mol Cell Cardiol. 2016;93:149–55. 26593722 10.1016/j.yjmcc.2015.11.015 PMC4846552

[69] Cancer BCECotNQCCf, et al. Expert consensus on the clinical application of PI3K/AKT/mTOR inhibitors in the treatment of breast cancer (2025 Edition). Cancer Innov. 2025;4(3):e70008. 40206206 10.1002/cai2.70008 PMC11981814

[70] Garg P, et al. Strategic advancements in targeting the PI3K/AKT/mTOR pathway for breast cancer therapy. Biochem Pharmacol. 2025;116850:p. 10.1016/j.bcp.2025.116850 40049296

