# Supplementary figures and images for: Multi-omics approach reveals CCND1, GABPA, HIF1A, and SOX6 as key regulators and prognostic markers in heart failure
Source: Hereditas. 2025 Aug 16;162:165. doi: 10.1186/s41065-025-00536-y (PMC12357434; doi:10.1186/s41065-025-00536-y)

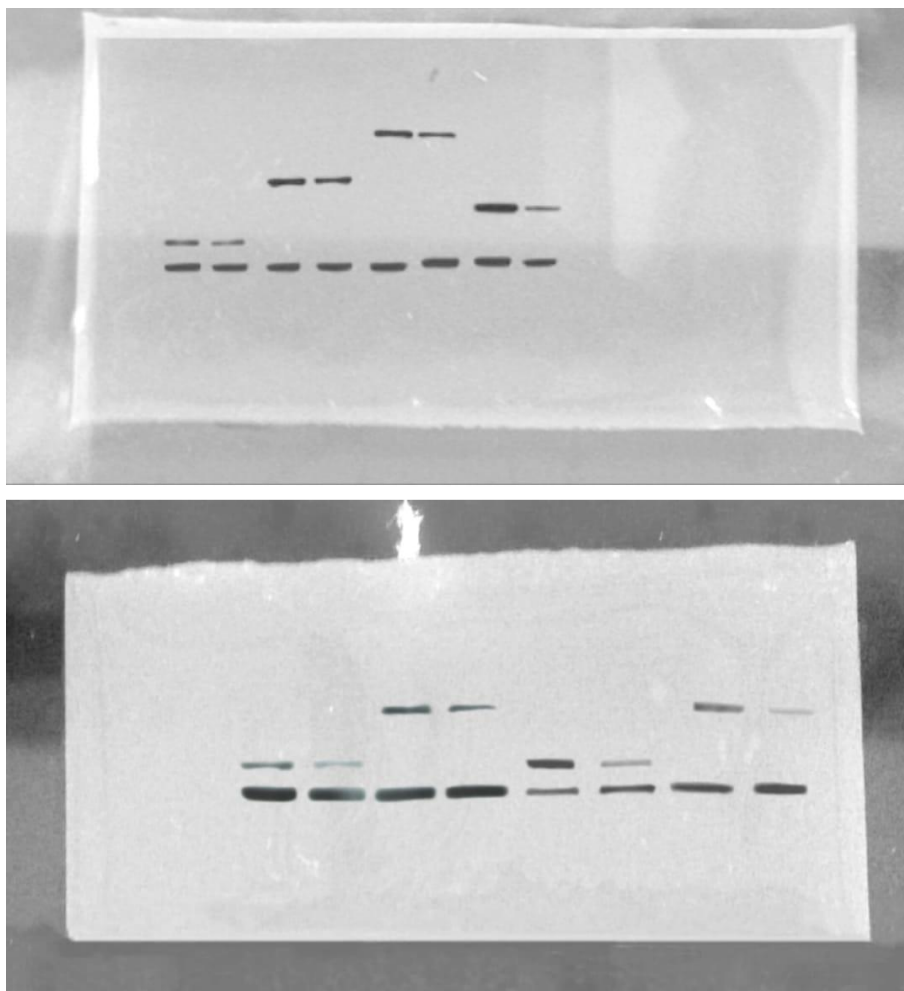

**Supplementary data Figure 1: Uncut Western blot bands of CCND1, GABPA, HIF1A, SOX6 and GAPDH.**

Supplement: Supplementary file 1 — Supplementary Material 1 [file 41065_2025_536_MOESM1_ESM.pdf]
